# Minimum acceptable diet feeding practice and associated factors among children aged 6–23 months in east Africa: a multilevel binary logistic regression analysis of 2008–2018 demographic health survey data

**DOI:** 10.1186/s13690-022-00882-7

**Published:** 2022-04-28

**Authors:** Misganaw Gebrie Worku, Tesfa Sewunet Alamneh, Getayeneh Antehunegn Tesema, Adugnaw Zeleke Alem, Zemenu Tadesse Tessema, Alemneh Mekuriaw Liyew, Yigizie Yeshaw, Achamyeleh Birhanu Teshale

**Affiliations:** 1 Department of Human Anatomy, College of Medicine and Health Science, School of Medicine, University of Gondar, Gondar, Ethiopia; 2 Department of Epidemiology and Biostatistics, Institute of Public Health, College of Medicine and Health Sciences, University of Gondar, Gondar, Ethiopia; 3 Department of Human Physiology, College of Medicine and Health Science, School of Medicine, University of Gondar, Gondar, Ethiopia

**Keywords:** Minimum acceptable diet, Children, Multilevel analysis, East Africa

## Abstract

**Background:**

Despite the proportion of receiving a minimum acceptable diet (minimum meal frequency and minimum dietary diversity) is lower in east Africa, there is limited evidence on minimum acceptable diet. Therefore, this study aimed to investigate the minimum acceptable diet and associated factors among children aged 6–23 months in east Africa.

**Methods:**

A secondary data analysis of the most recent Demographic and Health Survey (DHS) data of 12 east African countries was done. A total weighted sample of 34, 097 children aged 6–23 months were included. A multilevel binary logistic regression model was applied. The Intra-class Correlation Coefficient (ICC) and Median Odds Ratio (MOR) were calculated to assess the clustering effect. Besides, deviance was used for model comparison as the models are nested models. Both crude and adjusted Odds Ratio (OR) with a 95% Confidence Interval (CI) were reported as potential predictors of minimum acceptable diet feeding practice.

**Results:**

The prevalence of minimum acceptable diet feeding practice among children in east Africa was 11.56%; [95%CI; 11.22%, 11.90%]. In the multilevel analysis; child age of 12–17 month (AOR = 1.33: 95%CI; 1.20, 1.48), maternal primary (AOR = 1.21: 95%CI; 1.08, 1.35), secondary (AOR = 1.63: 95%CI; 1.44, 1.86) higher (AOR = 2.97: 95%CI; 2.30, 3.38) education level, media exposure (AOR = 1.38, 95%CI; 1.26, 1.51), household wealth statues (AOR = 1.28, 95%CI; 1.15, 1.42 for middle and AOR = 1.50: 95%CI; 1.42, 1.71 foe rich), employed mother (AOR = 1.27: 95%CI; 1.17, 1.37), maternal age 25–34 (AOR = 1.20: 95%CI; 1.09, 1.32) and 35–49 (AOR = 1.22: 95%; 1.06, 1.40) years, delivery in health facility (AOR = 1.43: 95%CI; 1.29, 1.59) and high community education level (AOR = 1.05: 95%CI; 1.01, 1.17) were positively associated with minimum acceptable diet child feeding practice. Meanwhile, the use of wood (AOR = 0.72: 95%CI; 0.61, 0.86) and animal dug (AOR = 0.34: 95%CI; 0.12, 0.95) as a source of cooking fuel and being from female-headed households (AOR = 0.88: 95%CI; 0.81, 0.96) were negatively associated with minimum acceptable diet feeding practice.

**Conclusion:**

Child age, mother’s educational level, source of cooking fuel, exposure to media, sex of household head, household wealth status, mother working status, age of the mother, place of delivery and community-level education were the significant determinants of minimum acceptable diet feeding practices. Therefore, designing public health interventions targeting higher-risk children such as those from the poorest household and strengthening mothers’ education on acceptable child feed practices are recommended.

## Background

Childhood undernutrition is a major public health problem, particularly in developing countries [1]. Globally, around 45% of infant and young child deaths occur due to malnutrition, where two-thirds of these are because of inadequate child feeding and associated infectious disease [1–4]. According to recent studies from low and middle income (LMICs) Countries, the magnitude of minimum acceptable diet among children ranges from 6.1% to 36% [5, 6]. The World Health Organization (WHO) and the United Nations Children’s Fund (UNICEF) recommends sufficient, safe and adequate complementary foods for children aged 6–23 months to meet their nutritional requirement and developmental needs [7, 8]. However, reports from low and middle-income countries indicated that many infants and young children are not receiving appropriate complementary foods [7, 9, 10]. Continued breastfeeding beyond six months should be supplemented by these complementary foods, as breast milk alone is not sufficient to fulfill their nutritional requirements [11]. As the first two years of life are a crucial window for ensuring optimum child growth and development, nutritional deficiencies during this period often contribute to impaired cognitive development, educational achievement and poor economic performance [10].

Appropriate breastfeeding practices and successful complementary feeding prevent the occurrence of various pathological conditions in the infant including; childhood under-nutrition[12], diarrheal disease[13, 14] and under-five mortality [12, 15]. Despite the demonstrated benefits of complementary feeding practice to the health and development of children, insufficient complementary feeding is still widespread in many developing countries [15, 16]. Hence, considering the minimum acceptable diet, which is a combination of minimum nutritional diversity and minimum meal frequency, as one of the main complementary feeding indicators [5], the WHO has established guidelines for infant and young child feeding practices. Therefore, promoting breastfeeding and appropriate complementary child feeding practices are very crucial for decreasing the above-mentioned consequences [17–19].

According to the finding of previous literature educational status, wealth status, media exposure, occupation, source of cooking fuel, place of delivery, antenatal care (ANC) visit and community-level education are associated with minimum acceptable diet feeding practice among children aged 6–23 months [5, 9, 17, 20–24].

Although identifying the potential determinants of infant and young child feeding practice will help to improve the conditions for child feeding and child nutrition status, there is insufficient updated information on the magnitude and determinants of minimum appropriate diet feeding practices in low-income countries. Therefore, this study aimed to determine the prevalence and associated factors of minimum acceptable feeding practices among children in east Africa.

## Methods

### Data source

Secondary data analysis was conducted based on the pooled data from the most recent Demographic and Health Surveys of east African countries conducted from 2008 to 2018 (Burundi_2016, Ethiopia_2016, Comoros_2012, Uganda_2016, Rwanda_2014/15, Tanzania_2015/16, Mozambique_2011, Madagascar_2008, Zimbabwe_2013/14, Kenya_2014, Zambia_2018, and Malawi_2015/16). Each country’s DHS survey consists of men, women, children, birth, and household datasets and the kids dataset (KR file) was used for this study. In the KR file, all children aged 6–23 months were considered for the analysis. The DHS used two stages stratified sampling technique to select the study participants. We pooled the most recent DHS surveys done in the 12 east African countries and a total weighted sample of 34, 097 was included in the final analysis. The total weighted sample of children included for each country was presented in Table 1.

**Table 1:** The study participants were included in the study by country and year of survey in east Africa

Country	Year of survey	Frequency (n)	Percentage
Burundi	2016	2,009	5.89
Ethiopia	2016	2,985	8.75
Kenya	2014	2,128	6.24
Comoros	2012	730	2.14
Madagascar	2008	1,427	4.19
Malawi	2015/16	1,556	4.56
Mozambique	2011	3,330	9.77
Rwanda	2014/15	2,352	6.90
Tanzania	2015/16	6137	18.00
Uganda	2016	2,845	8.34
Zambia	2018	5,484	16.08
Zimbabwe	2013/2014	3,114	9.13
Total		34,097	

### Variables of the study

#### Dependent variable

The minimum acceptable diet feeding practice was the outcome variable. It is a binary outcome variable, which was coded as 0 if the child didn’t feed a minimum acceptable diet and 1 if the child feed a minimum acceptable diet. The child is said to be fed with MAD if he/she had both minimum meal frequency and minimum dietary diversity in both breastfeeding and non-breastfeeding children. These children who received solid, semi-solid or soft foods, two times for breastfed infants 6–8 months, three times for breastfed children 9–23 months and four times for non-breastfed children is said to have minimum meal frequency. These children with 6–23 months of age received foods from four or more food groups of the seven food groups (Cereals, Legumes and nuts, Dairy products, Eggs, Flesh foods, Vitamin A-rich fruits and dark green leafy vegetables and other fruits) are said to have minimum dietary diversity [25].

#### Independent variables

The independent variables included in this study were respondent’s age, preceding birth interval, birth order, age of the child, sex of household head, family size, number of under-five children, maternal educational level, source of cooking fuel, distance to get water, media exposure, household wealth status, employment status, place of delivery, number of antenatal visits, residence, community poverty level, community educational level and community level of ANC utilization.

### Data management and analysis

Data extraction, recoding and analysis were done using STATA version 14 software. To restore the representativeness of the data as well as to get a reliable estimate and standard error, the data were weighted before doing any statistical analysis. The hierarchical nature of the DHS data, which violates the independent assumptions of the standard logistic regression model was handled with a multilevel logistic regression analysis. Children in the same cluster are more likely to be similar to each other than children from another cluster. This implies that there is a need to take into account the cluster variability by using advanced models such as the multilevel logistic regression model. The Interclass Correlation Coefficient (ICC) and Median Odds Ratio (MOR) were checked to assess whether there was clustering or not. Model comparison was done using deviance (-2LL). The null model-a model without explanatory variables, model I-a model with individual-level factors, model II-a model with community-level factors and model III-a model with both individual and community-level factors were fitted. Multicollinearity among the independent variables was checked using VIF and the mean VIF was less than 5, which indicates there is no multicollinearity among the included independent variables. Both bivariable and multivariable multi-level logistic regression were done. At the bivariable analysis variables with a *p*-value ≤ 0.2 were considered for multivariable analysis. In the multivariable multilevel analysis, the Adjusted Odds Ratio (AOR) with 95% Confidence Interval (CI) was reported to declare the statistical significance of the association.

## Results

### Individual and community-level characteristics of the study participants

Nearly 35% of children were in the age group of 12–17 months. The majority of the children (88.22%) were born within more than 24 months of pregnancy interval. Regarding media exposure, more than two-thirds (65.82%) of the mothers were exposed to at least one of the media sources (watching television, listening to the radio or reading a newspaper) and nearly half of households (46.29%) travel 30 min or longer round trip to fetch drinking water. Regarding the source of cooking fuel, only 3.26% were used electricity as a source of cooking fuel. More than half (55.96%) of the mothers had at least 4 ANC visits during their last pregnancy. About 46.11% of the children were from poor households and 52.28% of the mother had a primary education level. More than half (50.3%) of the mothers were from a community with a high poverty level. Nearly half of the mothers (50.49%) were from the community with high ANC services utilization (Table 2).

**Table 2:** Individual and community-level characteristics of the study participants in east Africa using 2008–2018 demographic health survey data (*N* = 34, 097)

Variables	Categories	Frequency	Percentage
Age of the child(months)	6–8	6006	17.61
	9–11	5829	17.10
	12–17	11,911	34.93
	18–23	10,351	30.36
Respondents age (years)	15–24	12,253	35.984
	25–34	15,367	45.07
	35–49	6475	18.99
Birth order	First	8012	23.50
	2^nd^-4^th^	16,560	48.57
	Fifth and above	9525	27.94
Preceding birth interval	Less than 24 month	4018	11.78
	24 months and above	3007	88.22
Number of antenatal care visits	0	2083	6.11
	1–3	12,935	37.94
	≥ 4	19,097	55.96
Family size	< 5	16,766	49.17
	≥ 5	17,331	50.83
Source of cooking fuel	Electricity	1111	3.26
	Charcoal	6130	17.98
	Wood	24,398	71.55
	Animal dug	178	0.52
	Other	2280	6.69
Number of under fifth children	No	384	1.12
	One	12,616	37.00
	More than one	21,098	61.88
Media exposure	No	11,654	34.18
	Yes	22,443	65.82
Wealth status	Poor	15,723	46.11
	Middle	6626	19.43
	Rich	11,747	34.5
Maternal educational	No education	7393	21.68
	Primary education	17,827	52.28
	Secondary education	7865	23.07
	Higher education	1012	2.97
Working statues	No	20,427	59.91
	Yes	13,670	40.09
Residence	Urban	8182	23.99
	Rural	25,916	76.01
Distance to a water source	30 min and less	19,744	53.29
	Greater than 30 min	17,308	46.71
Sex of household head	Female	7718	22.64
	Male	26,379	77.36
Community level education	Low	17,182	50.39
	High	16,915	49.61
Place of delivery	Home	8085	23.71
	Health facility	26,012	76.29
Community level poverty	Low	17,371	50.95
	High	16,726	49.05
Community level ANC utilization	Low	17,759	52.08
	High	16,338	47.92

### Prevalence of minimum acceptable diet feeding practice

**Fig. 1:**
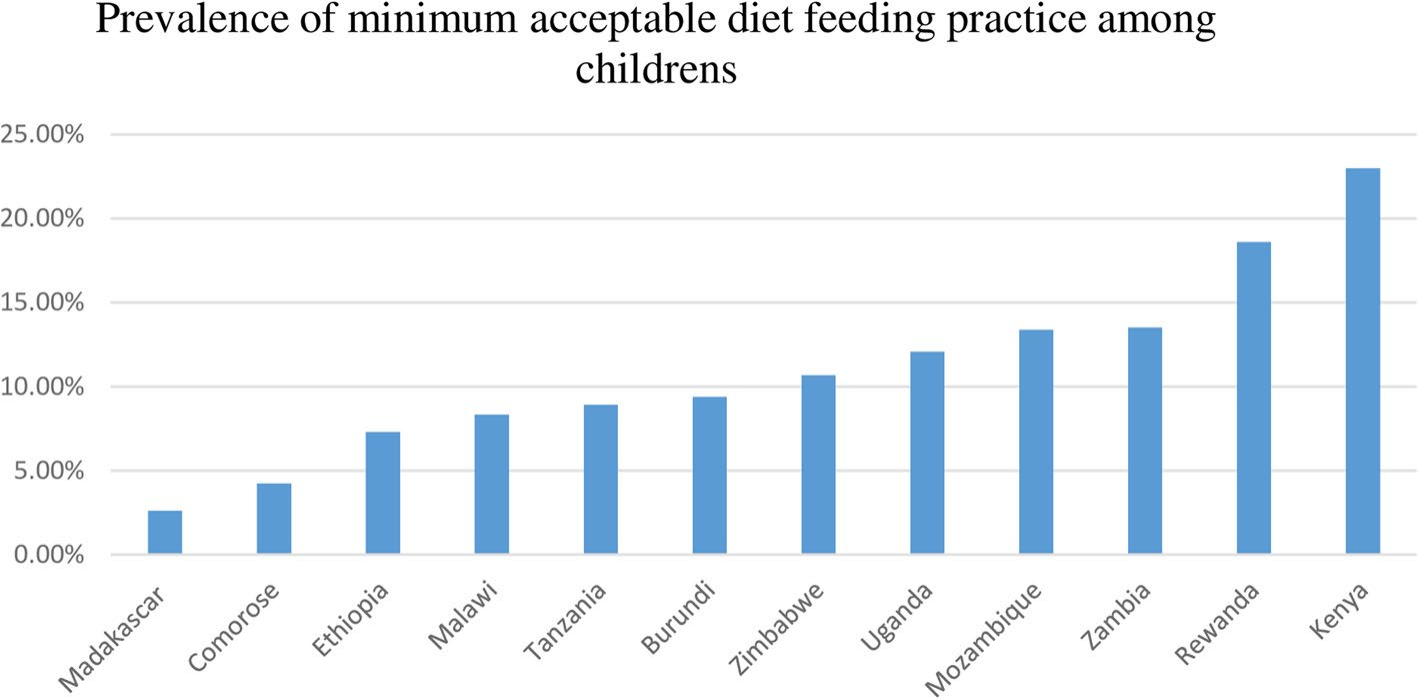
Showing the prevalence of minimum acceptable diet feeding practice in east Africa

The prevalence of minimum acceptable diet child feeding practice was 11.56% [95%CI: 11.22, 11.90] in east Africa. It was highest in Kenya (23%) and lowest in Madagascar (2.61%) (Fig. 1).

### Random effect model and model fitness

The ICC, MOR, and percentage change in variation (PCV) were used to assess the random-effect model of null model, model I, model II and model III. The ICC value of 0.069 in the null model indicates that a 6.9% variance in minimum acceptable diet feeding practice was due to cluster/community variations. In addition, the highest MOR value of 1.59 suggests a significant clustering of MAD feeding among children. In addition, the highest PCV (0.41%) in the final model (model III) indicated that both individual and community-level variables explained about 41% of the variation in minimum acceptable diet feeding practice. The final model (model III), which incorporates both individual and community level variables was the best-fitted model (it had the lowest deviance) (Table 3).

**Table 3:** Multilevel random effect model and model fitness of null model (a model without explanatory variables), model I (a model with individual-level factors), model II (a model with community level factors) and modell III (a model with both individual and community-level factors) for the assessment of minimum acceptable diet feeding practice among children of 6–23 months in eastern Africa using 2008–2018 demographic health survey data

Parameter	Null model	Model I	Model II	Model III
ICC	0.069	0.07	0.064	0.067
PCV	Reff	0.042	0.083	0.41
MOR	1.59	1.23	1.21	1.57
Model comparison				
Log likelihood	-12,006.411	-11,416.813	-11,838.149	-11,410.956
Deviance	24,012.822	22,833.626	23,676.298	22,821.912

*ICC* Intraclass Correlation Coefficient, *PCV* Percentage Change in Variation, *MOR* Median Odd Ratio

### Factors associated with minimum acceptable diet feeding

To determine the associated factors of MAD feeding practice, those variables with p ≤ 0.2 at bivariable analysis were entered to multivariable multi-level regression analysis. Accordingly, child age, mother’s educational level, source of cooking fuel, media exposure, sex of household head, household wealth status, mother working status, age of respondent, ANC visit and community-level education were independent predictors of MAD feeding (p ≤ 0.05). The odds of feeding a child with MAD was 1.33 times (AOR = 1.33: 95%CI; 1.20, 1.48) higher among children aged between 12–17 months than children aged 6–8 months. The odds of feeding the child with a minimum acceptable diet was 1.27 times (AOR = 1.27: 95%CI; 1.17, 1.37) higher among employed mothers compared with their counterparts. Mothers with media exposure had 1.38 times (AOR = 1.38; 95%CI; 1.26, 1.51) higher odds of feeding a MAD for their children than mothers who had no access to media. Mothers with primary (AOR = 1.21: 95%CI; 1.08, 1.35), secondary (AOR = 1.63: 95%CI; 1.44, 1.86) or higher (AOR = 2.97: 95%CI: 2.30, 3.38) educational level had higher odds to feed their children with MAD compared with mothers with no formal education. Old-aged mothers (AOR = 1.20: 95%CI; 1.09, 1.32 for 25–34 aged mothers and AOR = 1.22: 95%CI; 1.06, 1.40 for 35–49 aged mothers) were more likely to practice minimum acceptable diet feeding compared to young aged mother. Children from the female-headed household were less likely to meet the minimum acceptable diet (AOR = 0.88: 95%CI; 0.81, 0.96). Mothers who have used wood (AOR = 0.72: 95%CI; 0.61, 0.86), animal dug (AOR = 0.34: 95%CI; 0.12, 0.95) and other (AOR = 0.72: 95%CI; 0.59, 0.89) as a source of fuel were less likely to provide minimum acceptable diet to their child compared with those who used electricity as a source of fuel. Mothers who have delivered in the health facility had 1.43 times (AOR = 1.43: 95%CI; 1.29, 1.59) more likely to provide a minimum acceptable diet for their child compared with their counterparts. Regarding community-level education, mothers from the community of higher educational level were 1.05 times (AOR = 1.05: 95% CI; 1.01, 1.17) more likely to complement a minimum acceptable diet for their children (Table 4).

**Table 4:** The bi-variable and multivariable multilevel binary logistic regression analysis of factors associated with minimum acceptable diet feeding practice in east Africa using 2008–2018 demographic health survey data

Variables	Category	Minimum acceptable diet		Crude Odds Ratio(95%CI)	Adjust Odds Ratio (95%CI)
		Yes	No		
Age of the child (months)	6–8	634	5372	1	1
	9–11	609	5220	1.02(0.90, 1.15)	1.01(0.89, 1.14)
	12–17	1550	10,362	1.32(1.20, 1.47)	1.33(1.20, 1.48)*
	18–23	1148	9202	1.11(1.01, 1.23)	1.07(0.96, 1.20)
Respondents age (years)	15–24	1250	11,003	1	1
	25–34	1959	13,410	1.24(1.15, 1.34)	1.20(1.09, 1.32)*
	35–49	733	5743	1.12(1.02, 1.24)	1.22(1.06, 1.40)*
Birth order	1	1021	6991	1	1
	2–4	2008	14,552	0.92(0.85, 1.01)	0.96(0.87, 1.07)
	≥ 5	912	8613	0.73(0.66, 0.81)	0.98(0.84, 1.14)
Preceding birth interval (months)	< 24	330	3689	1	1
	≥ 24	3612	26,467	1.27(1.13, 1.42)	1.09(0.97, 1.23)
Number of ANC visit	0	145	1938	1	1
	1–3	1479	11,456	1.99(1.63, 2.43)	1.16(0.94, 1.44)
	≥ 4	2317	16,762	2.16(1.77, 2.63)	1.04(0.84, 1.29)
Family size	< 5	2037	14,729	1	1
	≥ 5	1904	15,427	0.88(0.82, 0.94)	0.96(0.88, 1.04)
Source of cooking fuel	Electricity	284	827	1	^1^
	Charcoal	1040	5090	0.62(0.53, 0.72)	0.86(0.73, 1.01)
	Wood	2264	22,134	0.30(0.26, 0.35)	0.72(0.61, 0.86)*
	Animal dug	7	171	0.12(0.04, 0.33)	0.34(0.12, 0.95)*
	Other	346	1934	0.45(0.37, 0.55)	0.72(0.59, 0.89)*
Number of under fifth children	No	51	333	1	1
	One	1681	10,935	1.12(0.82, 1.54)	1.06(0.75, 1.53)
	More than one	2209	18,888	0.84 (0.62, 1.16)	0.98(0.69, 1.40)
Media exposure	No	815	10,839	1	1
	Yes	3127	19,317	2.11(1.95, 2.29)	1.38(1.26, 1.51)*
Wealth status	Poor	1231	14,493	1	1
	Middle	684	5942	1.50(1.36, 1.67)	1.28(1.15, 1.42)*
	Rich	2027	9721`	2.60(2.40, 2.81)	1.5(1.42, 1.71)*
Maternal education level	No education	522	6871	1	1
	Primary education	1801	16,027	1.55(1.39, 1.72)	1.21(1.08, 1.35)*
	Secondary education	1310	6555	1.63(2.36, 2.94	1.63(1.44, 1.86)*
	Higher education	309	703	6.31(5.35, 7.44)	2.97(2.30, 3.38)*
Working statues	No	2546	17,882	1	1
	Yes	1396	12,274	1.31(1.22, 1.41)	1.27(1.17, 1.37)*
Residence	Urban	1479	6702	1	1
	Rural	2462	23,454	0.49(0.46, 0.53)	0.94(0.84, 1.04)
Distance to water source	≤ 30 min’	1898	16,415	1	1
	> Greater than 30 min’	2043	13,741	1.33(1.24, 1.43)	1.06(0.98, 1.14)
Sex of household head	Female	836	6883	0.90(0.83, 0.98)	0.88(0.81, 0.96)*
	Male	3106	23,273	1	1
Place of delivery	Home	531	7554	1	1
	Health facility	3410	22,602	2.09(1.90, 2.31)	1.43(1.29. 1.59)*
Community-level education	Low	1926	15,256	1	1
	High	2016	14,900	1.22(1.11, 1.35)	1.05(1.01, 1.17)*
Community-level poverty	Low	2077	15,294	1	1
	High	1864	14,863	0.87(0.79, 0.96)	1.17(1.00, 1.29)
Community-level ANC utilization	Low	1966	15,793	1	1
	High	1976	14,363	1.14(1.03, 1.26)	1.04(0.94, 1.16)

*ANC* Antenatal care, *CI* Confidence interval

^*^p-value < 0.05, **other = *lpg, natural gas, biogas, kerosene, coal, lignite, agricultural crop, straw/shrubs/grass, other,*

## Discussion

This study aimed to determine the minimum acceptable diet feeding practice and associated factors among children in east Africa. Accordingly, the prevalence of minimum acceptable diet child feeding practice in the region was 11.56% [95%CI = 11.22%, 11.90%]. The prevalence of minimum acceptable diet feeding practices in this study was higher than the findings of other studies [5, 26, 27]. The prevalence was lower than reports in Africa and Asia [20, 28].

In the multilevel multivariable analysis factors such as the age of the child, respondent age, source of cooking fuel, exposure to media, household wealth status, mother educational level, working status, sex of household head, place of delivery and community-level education were significantly associated with feeding minimum acceptable diet. Children with age of 12–17 months had higher odds to feed a minimum acceptable diet than a child with ages 6–8 months. This finding is in agreement with the study done in Ethiopia [5], Ghana [20], Uganda [21] and Indonesia [22]. This may be attributed to the late introduction of complementary feeding and the start of complementary feeding with only limited items (only milk or cereal). Mothers might also be able to perceive that the younger the children, the weaker the intestine’s capacity to digest such foods as banana, eggs, pumpkin, carrots, green vegetables and meat [29]. This may be further justified by traditional beliefs and practices, when introducing complementary food to infants, they may develop diarrhea due to poor hygienic conditions, but mothers may equate this problem with taking new food items and ultimately they would not encourage the child to eat foreign foods.

Similarly, employed mothers had higher odds to provide a minimum acceptable diet for their children. This finding is supported by studies conducted in Ethiopia [5, 30] and Serilanka [31]. This may be related to the earning capacity of the mother, which is an important factor in feeding the child with an appropriate diet. Increased access to resources, broader social networks and increasing awareness of their social environment could also improve the chances of feeding the child with the minimum appropriate diet [5]. Similarly, older aged women had a higher chance of providing MAD to their children compared with young women. This finding was supported by another study done among Indian population [32]. Such significant effects of maternal age on complementary feeding practice suggest that the mother’s experience may play a significant role inappropriate infant and young child feeding practices [32]. In this study exposure to public media was a significant predictor of feeding a minimum acceptable diet, which is in line with another study [9]. This might be associated with the influence of media exposure on behavioral change to improve the complementary feeding practice through enhancing mothers’ knowledge on feeding a minimum acceptable diet to their children [5].

The mother’s education level was significantly associated with feeding the child a minimum acceptable diet. A similar finding was reported from studies in Ethiopia [9], Tanzania [30], Ghana [20] and east Africa [23]. This may be related to higher maternal education improving the job opportunity of mothers and the decision-making process of households, which in turn is correlated with an improvement in the use of health services [33]. Similarly, higher household wealth statuses were significantly associated with minimum acceptable diet feeding practice. This finding was in agreement with another study done elsewhere [15, 23, 30]. The present study found that birthing in a health facility was significantly associated with higher odds of minimum acceptable diet feeding practice and this finding is supported by a study done in west Africa [24]. This might be as institutional delivery increases exposure to health information and improves mothers’ knowledge about infant and young child feeding [34]. In this study, children from a household who used traditional biomass as a source of fuel (wood, animal dug and others) had a lower chance to feed a minimum acceptable diet. This might be associated with improving access to affordable and reliable modern forms of energy services is essential, especially for developing countries in reducing poverty and promoting economic development [35]. Children from communities of higher educational level had more odds to be fed a minimum acceptable diet than children from the community of lower educational level and this finding is supported by the studies conducted in Tanzania [30] and east Africa [23]. This may also be because trained mothers were more likely to have sufficient knowledge, easy to understand the practice of child feeding, received lessons in school on child feeding that would improve their comprehension of the value of child feeding [5].

### Strength and limitations of the study

This study was based on nationally representative data with large sample size. Besides, it was based on an appropriate statistical approach (multilevel analysis) to accommodate the community or cluster level variability of minimum acceptable diet feeding. Moreover, since it is based on the national survey data the study has the potential to give insight to policymakers and program planners to design appropriate intervention strategies at the national level. However, this study had limitations in that the DHS is mostly based on respondents’ self-report and might have the possibility of recall bias.

## Conclusion

In this study, the prevalence of minimum acceptable diet feeding practices in eastern Africa was found to be below. Both individual and community-level factors were associated with minimum acceptable diet feeding practice. Child age, mother’s educational level, source of cooking fuel, media exposure, sex of household head, household wealth status, mother working status, age of the mother, place of delivery and community level of education were the significant determinants of minimum acceptable diet feeding practices. Therefore, giving special attention to children aged 6–23 months and practicing appropriate feeding practices should be implemented to decrease devastating health problems of the child associated with inappropriate minimum acceptable diet feeding practice. Also, designing public health interventions targeting higher-risk children such as those from the poorest household and strengthening mothers' education on acceptable child feed practices are recommended.

## Data Availability

All result-based data are within the manuscript and the data set is available online and any one can access it from www.measuredhs.com.

## Abbreviations

CI: Confidence Interval
CSA: Central Statistical Agency
DHS: Demographic Health Survey
EA: Enumeration Area
ICC: Intraclass Correlation Coefficient
LLR: Likelihood Ratio
LMIC: Low and middle-income country
PCV: Proportional change in Variance
WHO: World Health Organization
